# Patterns of psychopathology and personal strengths among youth in psychiatric care

**DOI:** 10.1002/jcv2.70156

**Published:** 2026-08-06

**Authors:** Logan R. Cummings, Gil Noam, Alessandro S. De Nadai, Kathryn Parker, Patricia Allen, Julianne G. Wilner, Jillian M. Russo, Lauren O. Delman, Aishwarya Surendran, Emily G. Arnott, Dawn E. Sugarman, Alisa Busch, Alexander Kos, Fairlee C. Fabrett, Ralph Buonopane, Kristen L. Batejan, Erika C. Esposito, Tian Yu Cheng, Samuel J. Marquis, Wilson Giles Stecher, Caroline S. Abut, Fiona Ritchie, Jeffrey Zhang, Emily A. Hutchinson, Isabella Kahhale, Hannah Becker, Jennifer T. Sneider, Marisa M. Silveri, Colin W. Burke, Elan French, Esther S. Tung, Abigail M. Stark, Jennifer R. Klix, Anna B. Mazur, Denice E. Cronin, Kayla M. Turner, Melanie R. Harkins, Peter Adams, Willow Clayton, Clare Foster, Dorothea R. Volpp, Emily Gaddy, Florence R. Stout, Alyssa L. Faro, Maria G. Fraire, Mary Gambill, Perihan Esra Guvenek‐Cokol, Christopher Alpert, Cynthia Kaplan, Erin V. Batarseh, Gillian Galen, Kesley A. Hobbs, Lola M. Rodriguez, Lukas A. Turlick, Rhea Agrawal, Sophia E. Nadeau, Allison P. Falls, Eliot Fearey, Daniella H. Levy, Ana L. Ibarra, Bryan C. Pridgen, Julia G. Luppino, Kelly Molthrop, Louise J. Weidner, Munya Hayek, Kayla J. Saucedo, Magdalena S. Batlle, Payton B. Maclean, Rajvi Broker‐Sen, Wendy P. Bamatter, Charles Moore, Rachel L. Wallace, Rae‐Anna Muise, Tessa N. Hamilton, Jacqueline B. Sperling, Paulina Loo, R. Meredith Elkins, Michelle C. Silverman, Daniel P. Dickstein

**Affiliations:** ^1^ Simches Division of Child and Adolescent Psychiatry McLean Hospital Harvard Medical School Belmont Massachusetts USA; ^2^ Mass General Brigham Department of Psychiatry Harvard Medical School Boston Massachusetts USA; ^3^ Department of Health Care Policy Harvard Medical School Boston Massachusetts USA

**Keywords:** adolescence, child, measurement‐based care, strengths‐based approaches, symptom assessment

## Abstract

**Background:**

The American Academy of Pediatrics and the American Academy of Child and Adolescent Psychiatry have declared a National State of Emergency in Children's Mental Health, calling for improved approaches to address youth mental health. We sought to leverage the first child psychiatry division‐wide measurement‐based care initiative to advance understanding of how both psychopathology and social‐emotional skills are related among youth patients, as these associations are rarely examined together. In doing so, our objective was to provide an empirical basis for integrative approaches that target psychopathology, while also leveraging social‐emotional strengths.

**Methods:**

Youth patients receiving a continuum of psychiatric services, from intensive outpatient to short‐term residential programs (*N* = 926; ages 11–24; 58% Cis‐Female; 84.6% White), completed self‐report measures of psychopathology and social‐emotional skills. Latent profile analyses were conducted to identify distinct profiles within the sample.

**Results:**

Seven profiles were identified. Profiles with overlapping syndromes were differentiated by highly distinct patterns of socio‐emotional strengths and challenges.

**Conclusion:**

Social‐emotional skills assessment provides unique information beyond syndrome severity and can be used to create personalized subgroups of clinical patients. We observed many youth with similar syndromes report markedly different patterns of social‐emotional skills. We emphasize the clinical utility of integrating strengths‐ and resilience‐based frameworks into psychiatric assessment and treatment planning—supporting the shift beyond treatment models focused solely on reducing symptoms and skills deficits, toward more personalized approaches that also leverage youths' existing capacities for growth and recovery.

## Key Points

**What's Known?**

- The prevalence of child and adolescent psychiatric disorders is a significant public health problem, necessitating innovative approaches to youth mental healthcare.
- Clinicians often take a deficits‐based approach, focusing on identifying and treating psychiatric syndromes that cause impairment, to the exclusion of integrating and leveraging a patient's inherent social‐emotional strengths that promote resilience and adaptive coping.

**What's New?**

- We conducted the first child psychiatry division‐wide measurement‐based care initiative to advance understanding of how both psychopathology and social‐emotional skills are related among youth patients, as these associations are rarely examined together
- We identified 7 distinct profiles, showing there is not a 1:1 relation between psychiatric syndrome severity and social‐emotional skills development

**What's Relevant?**

- Results highlight potential opportunities where clinicians could personalize existing evidence‐based treatments to target specific syndromes while leveraging youth’s social‐emotional strengths and capacity for resilience.

## INTRODUCTION

The incidence and prevalence of youth psychiatric disorders in the U.S. is a significant public health problem: psychiatric disorders onset before age 14 for 50% of the population, and before age 24 for 75% of the population (James et al., 2018). Moreover, suicide is the second leading cause of death for youth ages 10–24, with suicide deaths in this group increasing by 54% over the past two decades (CDC, 2025). Recently, the American Academy of Pediatrics (AAP), Child and Adolescent Psychiatry (AACAP), and Children's Hospital Association (CHA) declared a National State of Emergency in Children's Mental Health (*AAP‐AACAP‐CHA Declaration of a National Emergency in Child and Adolescent Mental Health*, n.d.) Late‐childhood through adolescence is a crucial period of social‐emotional development marked by substantial maturation of key brain systems involved in emotion regulation and social processing, facilitating significant shifts in interpersonal functioning (e.g., increased autonomy, greater peer influence, emergence of cliques and romantic relationships; Baker et al., 2025; Spear, 2000). This developmental period also coincides with the typical onset window for many mood and anxiety disorders, as symptoms frequently emerge during adolescence alongside major biological and hormonal changes (Black & Rofey, 2018; H. C. Meyer & Lee, 2019). Interventions that improve social‐emotional functioning during this developmental period have potential to significantly reshape trajectories of mental health as youth transition to adulthood (Dahl et al., 2018).

In response to calls by AAP, AACAP, and CHA, we examined the relationship between psychopathology and social‐emotional skills in a large (*N* = 926) sample of adolescents. As described in Kaplan et al. (2023), the Division of Child and Adolescent Psychiatry at McLean Hospital has implemented a division‐wide, measurement‐based care initiative (the “Child and Adolescent Routine Evaluation [CARE] Initiative”), in which every patient in every program has the opportunity to complete a standard battery of measures as part of routine clinical care. The current study is a collaborative effort between members of the Child and Adolescent Division, as well as support from hospital leadership (D.E.S, A.B.) and technical support (A.K.). The long‐term goal of this project is to provide an empirical basis for integrative approaches that target specific symptoms while leveraging a youth's unique social‐emotional strengths throughout treatment.

*Measurement‐based care* seeks to combine the art of clinical medical expertise with the science of standardized assessment, for more comprehensive diagnostic evaluations. By reducing the variability inherent in most of clinical medicine, which only relies on the clinical art of medicine (i.e., well‐trained empathic healthcare professionals), measurement‐based care seeks to improve outcomes, including recidivism, lack of remissions, and suicide. Current standard of care rarely uses standard assessments in any branch of healthcare. For example, studies show fewer than 17.9% of psychiatrists and 11.1% of psychologists routinely use any standardized assessments, such as symptom or functional impairment scales (Fortney et al., 2017). In contrast, the CARE Initiative uses standardized assessments collected from all youth patients receiving care across the division's 13 clinical programs. Although housed within a single division, the CARE Initiative is consistent with a multi‐site study, as our programs are located in 4 locations, spread over a 60‐mile radius, include highly specialized and more generalized programs, and provide care across the range of intensity from acute inpatient to residential, partial hospital, and outpatient.

Deficit‐based approaches, common in both physical and mental illness, focus on diagnosing and treating psychiatric syndromes (e.g., depression, worry/anxiety, irritability, anhedonia, sleep problems, distress, aggression) causing significant impairment in functioning. A deficit‐based approach is often the default approach to mental healthcare because it provides clear diagnostic criteria for a specific problem or change from typical health, facilitates access to treatment, and offers structured pathways for intervention (Blundo, 2001; Davis et al., 2024; Mathias et al., 2024). In particular, the Achenbach System of Empirically Based Assessment (ASEBA) is a well‐validated battery of questionnaires that include self‐, teacher‐, and caregiver‐report measures of internalizing disorder and externalizing disorder syndromes using criteria from the *Diagnostic and Statistical Manual of Mental Disorders, 5th Edition* (DSM‐5). The ASEBA system can detect risk and predict the onset of psychiatric disorders, which can inform interventions targeting specific syndromes (Deighton et al., 2014). For example, using ASEBA, Althoff and colleagues found the combination of elevated scores for attention problems, aggressive behavior, and anxiety/depression predicted the onset of anxiety and disruptive behavior disorders in adulthood (Althoff et al., 2010).

However, deficit‐based approaches have proven insufficient in mitigating the massive tide of youth mental health challenges. Increasing evidence suggests *strengths‐based approaches* may be a viable approach to not only reduce syndrome severity, but also promote factors associated with well‐being (e.g., increased feelings of belonging, autonomy and self‐efficacy, strengthening of social support networks [Cox, 2006; Davies et al., 2025; Lerner et al., 2005; Luebbe et al., 2024; Tse et al., 2016]). Strengths‐ and resilience‐based approaches focus on the identification and promotion of a child or adolescent's positive attributes, intrinsic skillsets, and positive relationships with peers and adults. Additionally, these approaches have greater emphasis on coping mechanisms and adaptive skills that help youth navigate current challenges and prepare for future stressors (Masten, 2018). One such measure of a youth’s perceived social‐emotional skill development is the Holistic Student Assessment (HSA). The HSA was developed based on the clover model for youth social‐emotional development, which organizes the development of specific social‐emotional skills into four broader domains (see Table 1; Malti & Noam, 2016; Noam et al., 2012).

**TABLE 1:** Definitions of social‐emotional domains and skills measured on the holistic student assessment (HSA).

Clover domain	Social‐emotional skill	Definition
*Action orientation*: Capacity to initiate action, seek challenges, and proactively shape one’s environment rather than passively responding to it	Action orientation	Engagement in physical and hands‐on activities
	Emotion control	Self‐regulation of distress and management of anger
*Assertiveness*: Capacity to express thoughts, needs, and feelings in direct, confident, and respectful ways	Assertiveness	Confidence in putting oneself forward, advancing personal beliefs, wishes, or thoughts and standing up for what one believes
	Perseverance	Persistence in work and problem‐solving despite obstacles
*Reflection*: Capacity to think critically about their experiences, emotions, and values	Reflection	Inner thought processes and self‐awareness and internal responsiveness toward broader societal issues
	Optimism	Enthusiasm for and hopefulness about one's life
*Belonging*: Capacity to form meaningful, trusting relationships and feel part of a community	Trust	Perception of other people as helpful and trustworthy
	Empathy	Recognition and understanding of others' feelings and experiences
	Relationships with peers and adults	Positive and supportive connections with friends, classmates, and adults

The HSA has been used in schools and community settings to identify unique areas of social‐emotional *strengths* (social‐emotional skills >1 standard deviation above the population mean) and *challenges* (social‐emotional skills >1 standard deviation below the population mean), which could inform the types of services and interventions young people receive (Malti & Noam, 2016). For example, in a large, school‐based sample, Malti et al. (2018) examined associations between social‐emotional skills and subscales on the Strengths and Difficulties Questionnaire (Goodman, 2001), a broadband self‐report measure assessing the severity of internalizing and externalizing problems, and prosocial behavior. Internalizing problems severity was associated with greater self‐reflection and empathy and lower optimism, assertiveness, action orientation, and emotion control on the HSA. Externalizing problems severity was associated with lower emotion control and reflection on the HSA. Noam et al. (2012) further demonstrated action orientation and assertiveness may co‐occur with elevated externalizing symptoms when not moderated by regulatory or relational skills, including emotion control, self‐reflection, and empathy. Their finding suggests a developmental pattern in which unbalanced social‐emotional skills (e.g., high assertiveness with low relational skills) may confer mental health risk.

Research with the HSA provides preliminary evidence of the complex interplay between youth psychopathology and social‐emotional skills—there is not an inverse relationship between symptom severity and an individual’s social‐emotional skill development. Findings by Noam et al. (2012) support clinical intuition that specific social‐emotional skills may be stronger among youth with specific psychiatric disorders. For example, it has been argued youth with attention‐deficit/hyperactivity disorder (ADHD) may have strengths in optimism, creativity, and assertiveness (Sherman et al., 2006). In support of these assumptions, evidence suggests youth with ADHD have similar levels of emotional intelligence as youth without ADHD, and variability in social‐emotional strengths among youth with ADHD is what fosters resilience in academic and family functioning (Charabin et al., 2023). Others have also found adolescents with elevated anxiet. also present with interpersonal strengths such as empathy and cooperation (Huttunen et al., 2025).

Nonetheless, there is a dearth of research on the associations between social‐emotional strengths and resiliencies associated with specific disorders. The HSA, and similar measures, have mostly been studied using non‐clinical samples and/or assessing broad syndrome domains (e.g., externalizing vs. internalizing symptoms). This research gap has strengthened a phenomenon known as the *clinician's illusion*, whereby inferences about a specific disorder are made based solely on a practitioner's own experience, without integration of information from other sources or research (Cohen & Cohen, 1984). Measurement‐based care could therefore mitigate the impact of the clinician's illusion in youth mental healthcare.

A more integrative approach, examining psychiatric syndromes and social‐emotional skills simultaneously, is important to advance our understanding of developmental psychopathology and resilience in youth with mental health challenges. Although the process of arriving at a psychiatric diagnosis should focus on atypical patterns in behavior as defined by the DSM‐5 that cause clinically significant impairment in one’s life (i.e., syndromes), it is essential for clinicians to also systematically assess for a youth’s potential socioemotional strengths, consistent with a measurement‐based approach, allowing for socioemotional strengths to be incorportated into a patient’s diagnostic formulation, case conceptualization, and treatment planning. We sought to build an empirical basis for this integrative approach by evaluating the relationship between syndromes and resilience in a large, multi‐site, sample of children and adolescents participating in our division's CARE Initiative (Kaplan et al., 2023). We used the ASEBA Self‐Report DSM‐5 oriented syndrome subscales (youth self‐report [YSR] for youth ages 11–18 years old, adult self‐report [ASR] for transitional‐aged youth over age 18) to measure psychiatric syndromes and the HSA to measure social‐emotional skill strengths/challenges. This approach moves beyond broader constructs of psychopathology (i.e., internalizing and externalizing disorder problems) to allow more granular mapping between DSM‐5 oriented syndromes and social‐emotional skills. We conducted a latent profile analysis (LPA) using both the YSR/ASR syndrome subscales and HSA social‐emotional skills subscales as indicator variables to detect idiographic constellations of co‐occurring syndromes and social‐emotional skills. Latent profile analysis is an ideal approach to our research questions because it is a data‐driven, person‐centered (vs. variable‐centered) approach that maximizes relations between individuals instead of variables, allowing for meaningful subgroups of individuals within a sample to be identified (Ferguson et al., 2020; Spurk et al., 2020).

In contrast to an inverse association between syndrome severity and social‐emotional strengths, we hypothesized profiles would emerge characterized by elevated syndrome severity and numerous social‐emotional strengths. We made three predictions based on the Clover Model of Social‐Emotional Development and studies using the HSA. First, youth with elevated internalizing syndromes would be characterized by strengths in empathy and self‐reflection, with challenges in action orientation, assertiveness, emotion control, and optimism. Second, youth with elevated externalizing syndromes would be characterized by strengths in assertiveness and action orientation, with challenges in emotion control, reflection, and empathy. Third, given many of the participants were receiving intensive psychiatric care due to severe suicidality or aggression, and given that belonging is central to prominent models of suicide (Van Orden et al., 2010) and developmental psychopathology models of nonsuicidal self‐injury (Cummings et al., 2021) and CD (Beauchaine et al., 2015), we predicted profiles with higher syndrome severity would be characterized by lower belonging.

## METHOD

### Procedure

Youth (11–24 years old) receiving clinical care in our division were offered the opportunity to participate in our Institutional Review Board‐approved, *CARE Initiative* (Kaplan et al., 2023). Although youth younger than 11 years participated in CARE, our broadband measure of psychopathology (ASR/YSR) is validated for youth 11 years or older. Therefore, we did not analyze participants younger than 11. Our division encompasses a continuum of care including adolescent acute inpatient, residential, short‐term residential (defined as 4–12 weeks of voluntary 24‐h clinical diagnostic and treatment services), partial hospital, and intensive outpatient. Our division also includes specialty programs providing dialectical behavioral therapy for adolescents, treatment of obsessive‐compulsive disorder (OCD), and more general programs. For patients who received multiple episodes of care during the study period, data from their first episode were included in the analysis. Data were collected from March 3, 2022 to February 18, 2025.

### Measures

#### Demographics

Participants used a drop‐down menu to select whether their sex assigned at birth was “male,” “female,” “intersex,” or “other.” Participants were asked “What is your current gender identity” and were provided a free‐text field to input their response. Participants indicated (yes or no) if they identified as “Hispanic and/or Latinx.” Participants were asked to indicate the race(s) with which they identify and were allowed to indicate more than one race.

#### Social‐emotional skills—holistic student assessment

The Holistic Student Assessment (HSA) (Noam et al., 2012) has 10 subscales assessing social‐emotional skills: *action orientation* (engagement in physical and hands‐on activities), *emotion control* (self‐regulation of distress and management of anger), *assertiveness* (confidence in putting oneself forward, advancing personal beliefs, wishes, or thoughts and standing up for what one believes), *perseverance* (persistence in work and problem‐solving despite obstacles), *optimism* (enthusiasm for and hopefulness about one's life), *reflection* (inner thought processes and self‐awareness and internal responsiveness toward broader societal issues), *trust* (perception of other people as helpful and trustworthy), *empathy* (recognition of others' feelings and experiences), *relationships with peers*, and *relationships with adults*. The HSA has been validated in diverse cultural and community samples of children and adolescents (Malti et al., 2018; Noam et al., 2012). Raw scores were converted into standardized scores (*z*‐scores; *M* = 0, SD = 1) using normative data stratified by age and sex assigned at birth. Z‐scores > 1 are considered a *normative social‐emotional strength* and *z*‐scores < 1 are considered a *normative social‐emotional challenge*.

#### DSM‐5 disorder‐oriented syndromes—[youth self‐report (YSR)]/[adult] self‐report (ASR)

Participants ages 11–18 completed the YSR (valid for ages 11–18) and those older than 18 completed the ASR (valid for ages 18–59). The YSR/ASR include DSM‐5 oriented subscales assessing the severity of syndromes of depression, anxiety, OCD, somatic concerns, and ADHD. The YSR includes a conduct disorder (CD) syndrome subscale, whereas this subscale is replaced by antisocial personality disorder on the ASR given diagnostic overlap and homotypic continuity between these two disorders (Whipp et al., 2019). The YSR/ASR yield standardized scores based on a population mean of 50 and a standard deviation of 10 (i.e., a *t*‐score) for each DSM‐5 subscale. For ease of interpretability and to reduce convergence errors when conducting the LPA (Sinha et al., 2021), we converted *t*‐scores on the YSR/ASR to *z*‐scores ([*t*‐score − 50]/10) prior to analyses; *z*‐scores >1 are considered elevated.

### Analytic strategy

We used the *tidyLPA* package in R v 4.2.3 to conduct latent profile analyses (Rosenberg et al., 2019). Models were estimated using YSR/ASR and HSA subscales as indicator variables, with indicator variances constrained to be equal and indicator covariances constrained to 0. As recommended by Spurk et al. (2020), we estimated a sequence of models (in our case, 1–12 classes) and assessed model fit by comparing the Bayesian Information Criterion (BIC) values for each model along with model entropy and interpretability.

Once class membership for each participant was obtained we conducted Pearson chi‐square tests and analysis of variance tests to determine whether demographic and clinical characteristics differed between profiles. Due to the low number of participants receiving intensive outpatient treatment (*n* = 15; 1.62%), cell sizes were too small for parametric comparisons on this variable, and these participants were removed from the chi‐square analysis.

## RESULTS

### Participant characteristics

Of the 1286 participants who consented to participate in CARE at the time this study was initiated, 28% were not included in the analysis because: they did not complete the HSA (*n* = 183), did not complete the YSR/ASR (*n* = 125), or did not complete enough items on either the HSA or YSR/ASR for a standardized score to be estimated (*n* = 52). As detailed in Table 2, participants in the final sample (*N* = 926) were 11–24 years old (*M* = 15.9, SD = 2.03), predominantly cisgender female, assigned female at birth, and White (see Supporting Information S1: Table S1). Participants excluded from analysis were not significantly different from participants included in analyses with respect to the distribution of sex, race, or Latinx identities as assessed by chi‐square tests. Participants excluded from analysis (*M* = 15.6 years old, SD = 2.2) were about 4 months younger than participants included in the analysis (*M* = 15.9 years old, SD = 2.0), Cohen’s *d* = 0.14, *t* = −2.20, *p* = .028; although statistically significant, we interpreted this age difference as having a negligible impact on the generalizability of our results. A chi‐square test indicated a significant association of missingness with program type, though the effect size was small; *χ* ^2^(4) = 32.5, *p* < .001, Cramer’s *V* = 0.16. Inspection of standardized residuals showed that partial hospitalization program participants were overrepresented in the final analytic sample (residual = 4.97) and residential (−2.33) and short‐term residential (−4.01) participants were underrepresented in the final analytic sample.

**TABLE 2:** Participant characteristics (*N* = 926).

	*n* (%)
Gender	
Cis‐female	542 (58.5%)
Cis‐male	215 (23.2%)
Nonbinary^a^	112 (12.1%)
Trans‐female	2 (0.2%)
Trans‐male	33 (3.6%)
Uncertain	22 (2.3%)
Gender minority	147 (15.8%)
Sex assigned at birth	
Female	673 (72.7%)
Male	253 (27.4%)
Level of care/placement	
Partial hospitalization	409 (44.2%)
Acute inpatient	197 (21.3%)
Residential	160 (17.3%)
Short‐term residential	145 (15.7%)
Intensive outpatient	15 (1.6%)
Race^b^	
American Indian or Alaska native	8 (0.9%)
Asian	104 (11.2%)
Black	43 (4.6%)
Native Hawaiian or Pacific Islander	4 (0.4%)
White	783 (84.6%)
Not listed	54 (5.8%)
Do not know	29 (2.1%)
Latinx/Hispanic	105 (11.3%)

^a^ Fourteen nonbinary gender identities were reported (see Supporting Information S1: Table S1). Nonbinary participants (*n* = 112) are reported separately from transgender participants (*n* = 35).

^b^ Proportions exceed 100% because 103 participants (11.1%) endorsed 2+ racial identities.

### Latent profile analysis

#### Global patterns

As detailed in Supporting Information S1: Appendix S1 (including Table S2 and Figures S1–S4), there were large improvements in model fit as the number of latent profiles increased from one to seven (ΔBIC > 150), but minimal improvements thereafter (ΔBIC < 60). Based on BIC values and qualitative interpretability, a 7‐profile model was retained.

**FIGURE 1:**
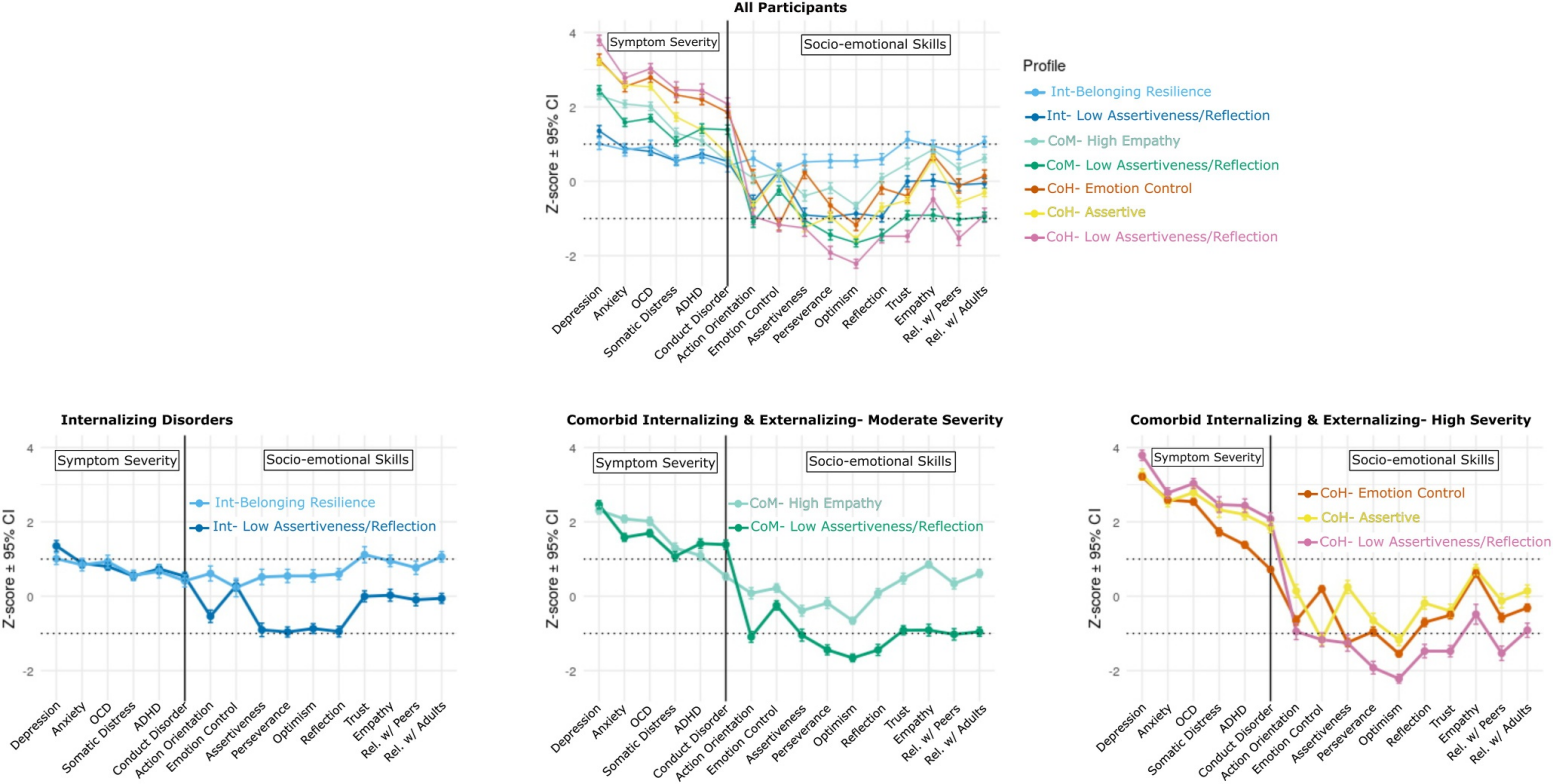
LPA yielded a 7‐profile solution, organized into 3 groupings: Internalizing (bottom left), comorbid internalizing and externalizing disorders—moderate severity (bottom center), and comorbid internalizing and externalizing disorders—high severity (bottom right). *z*‐scores ≥ 1 indicate syndrome severity is clinically elevated on the Y/ASR and a social‐emotional strength on HSA. *z*‐scores ≤ 1 indicate a social‐emotional challenge the HSA. ASR, adult self‐report; HSA, Holistic Student Assessment; LPA, latent profile analysis.

We observed a global pattern wherein groups of profiles had similar syndromes, but were characterized by distinctive patterns of social‐emotional skills. Furthermore, although we did not formally test whether profiles could be divided into subgroups, we observed three subgroups, as presented in Figure 1: *Internalizing Disorders* (2 profiles; bottom left), *Comorbid Internalizing and Externalizing Disorders with Moderate Severity* (2 profiles; bottom center), and *Comorbid Internalizing and Externalizing Disorders with High Severity* (3 profiles; bottom right). We did not find an inverse relationship between syndrome severity and social‐emotional skills. Rather, despite all participants requiring a level of care equal‐to‐or‐greater than intensive outpatient therapy, profiles were characterized by predominantly normative social‐emotional strengths/challenges, with profile‐specific patterns of social‐emotional strengths/challenges.

#### Profile‐specific patterns

We highlight notable observations guiding our discussion herein the manuscript. Additional fine‐grained examinations of each profile and potential clinical implications are detailed in Table 3 and Supporting Information S1: Table S3.

**TABLE 3:** Profile summaries and potential opportunities for treatment personalization.

Profile	Elevated syndromes	Normative challenges	Normative strengths	Belonging profile	Tiers composition		Relative challenge	Relative strengths	Opportunities for treatment personalization
Int—Low assertiveness/reflection	Depression, anxiety, OCD	Assertiveness, optimism, perseverance, reflection	None	*Typical*: All domains	Intervention (T3):	79.7%	Assertiveness, perseverance, reflection, optimism	Emotion Ctrl	Incorporate activities that emphasize practicing assertiveness, perseverance, and reflection during behavioral activation (e.g., when treating depression) or exposures (e.g., when treating anxiety).
					Prevention (T2):	13.6%			
					Promotion (T1):	3.8%			
Int—Belonging resilience	Depression, anxiety, OCD	None	Trust, empathy, Rel. w/Adults	*Typical*: Peers *High*: Trust, empathy, adults	Intervention (T3):	0.0%	Action orientation, emotion Ctrl, assertiveness, perseverance, optimism	Trust, empathy, Rel. W/Adults	Leverage high levels of trust and relationships with adults to facilitate therapeutic alliance and treatment buy‐in (e.g., during exposures for anxiety or OCD). Given relational strengths, group therapy, mentoring, or family‐based work might align well. Leverage empathy to improve interpersonal effectiveness skills.
					Prevention (T2):	2.7%			
					Promotion (T1):	97.3%			
CoM—High empathy	Depression, anxiety, OCD, somatic, ADHD	None	None	*Typical*: All domains	Intervention (T3):	32.5%	Assertiveness, perseverance, optimism	Trust, empathy, Rel. w/adults	Intervention could focus on enhancing emerging strengths (e.g., empathy, adult relationships) to buffer symptoms; may be more amenable to socio‐emotional learning or resilience‐focused approaches than profile D.
					Prevention (T2):	19.1%			
					Promotion (T1):	48.4%			
CoM—Low assertiveness/reflection	Depression, anxiety, OCD, somatic, ADHD, CD	All	None	*Low*: All domains	Intervention (T3):	100.0%	None	Emotion Ctrl	Leverage patients’ relative strength in emotion control when designing treatment plans. For example, patient may be more receptive to implementing distress tolerance skills than “change skills” in dialectical behavior therapy (DBT). Interventions should incorporate activities that foster belonging, which may support symptom reduction.
					Prevention (T2):	0.0%			
					Promotion (T1):	0.0%			
CoH—Emotion control	Depression, anxiety, OCD, somatic, ADHD	Assertiveness, perseverance, optimism	None	*Typical*: All domains	Intervention (T3):	93.8%	Assertiveness, optimism	Emotion Ctrl, empathy	Given challenges in assertiveness and optimism, interventions to boost perceived agency and positive outlook may have greatest impact on global functioning
					Prevention (T2):	5.0%			
					Promotion (T1):	1.2%			
CoH—Assertive	Depression, anxiety, OCD, somatic, ADHD, CD	Emo Ctrl, optimism	None	*Typical* all domains	Intervention (T3):	62.3%	Emo Ctrl, optimism	Assertiveness, empathy	Leverage typical levels of belonging to promote peer‐ and adult‐connectedness while targeting emotion control and optimism directly.
					Prevention (T2):	16.2%			
					Promotion (T1):	21.2%			
CoH—Low assertiveness/reflection	Depression, anxiety, OCD, somatic, ADHD, CD	All except empathy	None	*Typical*: Empathy *Low*: Trust and Rel w/Peers and adults	Intervention (T3):	100.0%	Perseverance, optimism	Empathy	Leverage empathy to build therapeutic alliance and treatment buy‐in. Given numerous social‐emotional challenges, especially relative challenges in perseverance and optimism, acceptance‐based approaches, may be more tolerable for patients. Given relative strength in empathy, mentalization‐based therapy may be effective.
					Prevention (T2):	0.0%			
					Promotion (T1):	0.0%			

*Note*: Normative strength = whole‐sample *z*‐score > 1; relative strength = within‐profile z‐score >1. Normative challenge = whole‐sample *z*‐score < 1; relative challenge = within‐profile z‐score < 1.

##### Internalizing disorder (Int) group

Syndromes were nearly identical, except depression syndrome severity. Although both profiles were characterized by normative social‐emotional skills in many domains, the profile with greater depression syndrome severity also was characterized by challenges in assertiveness, perseverance, optimism, and reflection, and no strengths, which we labeled as *Int‐Low Assertiveness/Reflection* (*n* = 119). The other profile was characterized by strengths in trust, empathy, and relationships with adults, and no challenges; hence we labeled this profile *Int with Belonging Resilience* (*n* = 74).

##### Comborbid internalizing and externalizing—moderate severity (CoM) group

Syndromes were similar among profiles in the CoM Group; however, one profile was characterized by elevated CD syndrome severity and one profile was not. Social‐emotional challenges were pervasive in the profile characterized by elevated CD syndrome severity, especially assertiveness, perseverance, optimism, and reflection. Therefore, we labeled this profile *CoM with Low Assertiveness/Reflection* (*n* = 154). Normative social‐emotional skills were observed in the profile without elevated CD syndrome severity, with relative strengths in belonging, especially empathy; hence, we labeled this profile *CoM with High Empathy* (*n* = 157).

##### Comorbid Internalizing and externalizing—high severity (CoH) group

Although syndromes were similarly elevated in the CoH group, they differed most with respect to depression and CD syndrome severity. Social‐emotional challenges were pervasive and especially low in perseverance and optimism in the profile characterized by the greatest syndrome severity. Therefore, we labeled this profile as *CoH‐ Low Assertiveness/Reflection* (*n* = 82). Two profiles were characterized by predominantly normative social‐emotional strengths/challenges, with relatively high levels of empathy, yet differed with respect to emotion control and assertiveness. We labeled the profile with normative emotion control (and challenge in assertiveness) as *CoH‐Emotion Control* (*n* = 242). Alternatively, we labeled the profile with normative assertiveness (and challenge in emotion control) *CoH‐Assertive* (*n* = 99).

### Sociodemographic differences between profiles

We did not find significant differences between profiles with respect to age (*F*(6,919) = 0.92, *p* = .477), the proportion of patients identifying as Latinx/Hispanic (*χ* ^2^(6) = 11.054, *V* = 0.11, *p* = .087), or Level of Care (*χ* ^2^(18) = 28.51, *V* = 0.10, *p* = .055).

Profiles differed with respect to sex assigned at birth (*χ* ^2^(6) = 23.80, *V* = 0.16, *p* < .001): There were fewer participants assigned female at birth (residual = −2.30) and more participants assigned male at birth (residual = 3.30) in the Int‐Low Assertiveness/Reflection profile, and fewer participants assigned male at birth (residual = −1.98) in the CoH‐Assertive profile than expected.

Profiles differed with respect to gender minority status (*χ* ^2^(6) = 44.21, *V* = 0.22, *p* < .001): there were fewer participants with gender minority identity in the Int‐Low Assertiveness/Reflection (residual = −2.92) and CoM‐High Empathy profiles (residual = −2.55) and more participants with gender minority identity in the CoH‐Low Assertiveness/Reflection profile (3.89) than expected.

## DISCUSSION

To the best of our knowledge, this study is the first to harness a Child/Adolescent Division‐wide measurement‐based care effort to advance what is known about the association between psychiatric syndromes and perceived social‐emotional strengths/challenges. Our primary finding, in this large youth sample (*N* = 926), spanning a socioeconomically diverse, 60‐mile catchment area, across four major sites, was identifying seven profiles, each with distinct patterns of psychiatric syndromes and social‐emotional strengths/challenges. Empathy appeared to be a relative strength within each profile. Social‐emotional skills that foster an individual’s Assertiveness/Reflection/motivation (action orientation, assertiveness, perseverance, optimism) and belonging (trust, empathy, relationships with peers and adults) tended to differentiate profiles.

Importantly, we found a complex interplay between psychiatric syndromes and resilience—it was not a simple inverse relationship of those with the greatest syndrome severity having the lowest resilience. As evident in Figure 1, profiles with nearly overlapping syndromes had divergent patterns of socio‐emotional strengths and challenges. Hence, our hypotheses were partially supported because socio‐emotional strengths/challenges diverged among profiles with similar syndromes. For example, internalizing syndrome severity was associated with challenges in assertiveness, perseverance, and optimism for the Int‐Low Assertiveness/Reflection profile, but not the Int‐Belonging Resilience. In contrast, internalizing syndrome severity was associated with strengths in trust, empathy, and relationships in the Int‐Belonging Resilience profile, but not the Int‐Low Assertiveness/Reflection profile. Consistent with our third hypothesis, profiles characterized by greater syndrome severity tended to have challenges with Belonging (CoM‐ Low Assertiveness/Reflection and CoH‐Low Assertiveness/Reflection), but not always (e.g., CoH‐Assertive).

Our findings underscore the importance of attending to not only psychiatric syndromes, but also social‐emotional strengths and challenges during assessment and treatment planning. As we elaborate in Table 3, systematically attending to a youth’s unique pattern of socio‐emotional strengths/challenges during the assessment phase may enhance treatment planning and personalization for youth with otherwise similar syndromes. For example, youth with an Int‐Belonging Resilience profile may benefit from interventions that leverage relational strengths, such as transdiagnostic, group‐based, cognitive‐behavioral interventions, which would allow youths to leverage social support and connectedness with same‐age peers to overcome challenging treatment obstacles (e.g., exposure hierarchies; Ehrenreich et al., 2009). In contrast, participating in group therapy (vs. individual therapy) may be less impactful for youth with a Int‐Low Assertiveness/Reflection profile. Instead, youth with Int‐Low Assertiveness/Reflection profile may benefit most from interventions that enhance one’s (cognitive or affective) capacity to engage in pleasant activities, such as behavioral activation (Dimidjian et al., 2011). Or, given potential ambivalence toward change, youth in the Int‐Low Assertiveness/Reflection profile may benefit most from transtheoretical (Hettema et al., 2005) or acceptance‐based interventions (Hayes et al., 2006). The divergence in socio‐emotional strengths/challenges between the CoH‐Empathy and CoH‐Assertive profiles also offers key insight for personalizing treatment intensity. Whereas both CoH‐Assertive and CoH‐Empathy profiles have nearly identical syndromes, CoH‐Empathy has many more socio‐emotional challenges than CoH‐Assertive, including challenges in reflection and belonging skills. Hence, whereas discharge to weekly outpatient therapy may be sufficient for youth with a CoH‐Assertive profile, youth with a CoH‐Empathy profile may require discharge to a more intensive, skills‐based treatment that teaches mindfulness, distress tolerance, and interpersonal effectiveness skills, and offers between‐session access to the clinician to help these skills to generalize to an adolescent’s daily experiences (e.g., dialectical behavior therapy [DBT]; McCauley et al., 2018). Additionally, as advocated by Noam et al. (2012), youth with unbalanced socio‐emotional skills may be at greater risk for psychiatric disorders (e.g., high assertiveness and low empathy may increase risk for externalizing disorders). In these instances, interventions could strive to balance leveraging existing strengths while also enhancing underdeveloped socio‐emotional skills.

Several themes emerged that align with developmental psychopathology models regarding the relationships between psychiatric syndromes and social‐emotional strengths/challenges. First, empathy was found to be a normative strength (2 profiles) or relative strength (3 profiles) in five of the seven profiles, consistent with biosocial theory in DBT, which posits individuals with high emotional sensitivity (i.e., empathy) may also be more vulnerable to emotion dysregulation (Crowell et al., 2009). Second, some profiles with prominent internalizing syndromes had difficulties with assertiveness, perseverance, and optimism (Int‐Low Assertiveness/Reflection, CoM‐Low Assertiveness/Reflection, CoH‐Low Assertiveness/Reflection), consistent with previous evidence that youth experiencing internalizing disorders may struggle with agency and hope, and consistent with cognitive‐behavioral theories of depression (Haaga et al., 1991) and anhedonia (Treadway & Zald, 2011) that posit pessimistic views of the self, the world, and the future lead to ineffective patterns of thinking and behavior. Third, profiles with more prominent externalizing syndromes—particularly those with elevated CD syndrome severity (CoM‐Low Assertiveness/Reflection, CoH‐Low Assertiveness/Reflection, CoH‐ Assertive)—tended to report lower emotion control and self‐reflection. This finding aligns research showing youth with low self‐awareness and poor emotion regulation—particularly with regulating negative emotions—are more likely to develop ADHD, oppositional defiant disorder, and CD (Graziano et al., 2022; Halligan et al., 2013; Ros & Graziano, 2018). Fourth, profiles characterized by low relational strengths (i.e., belonging) were characterized by especially high syndrome severity (CoM‐Low Assertiveness/Reflection, CoH‐Low Assertiveness/Reflection), consistent with interpersonal models of youth anxiety, depression (Silk et al., 2012), and externalizing disorders (Beauchaine et al., 2015). Relatedly, not only was Int‐Low Assertiveness/Reflection characterized by low belonging, but it was also over‐represented by participants who self‐identified as a gender minority, consistent with minority stress theory (I. H. Meyer, 2003)—that stigma, prejudice, and real/perceived social rejection exacerbates mental health challenges among sexual and gender minorities. Finally, 19% (*n* = 126) of the sample had more social‐emotional strengths than challenges, relative to their same‐age and gender peers, further underscoring psychopathology does not correspond one‐to‐one, in a linear fashion, with social‐emotional functioning. Our results demonstrate that consideration of social‐emotional strengths and resilience can be integrated into existing theoretical frameworks underpinning evidence‐based treatments for youth psychopathology.

Taken as a whole, our data highlight the value of measurement‐based [youth mental health] care. Our data suggest the need to better integrate standard assessment of psychopathology and social‐emotional strengths and resilience in clinical care for children and adolescents, which may guide treatment decisions (see Table 3) and catalyze treatment development in the future. Meta‐analytic reviews have found community‐, strengths‐based interventions focused on enhancing social‐emotional functioning and resilience factors—especially interventions based on the positive youth development theoretical framework (Lerner et al., 2005)—lead to better academic achievement and psychological adjustment (Ciocanel et al., 2016; Durlak et al., 2010). Hence, integrating strengths‐based approaches into standard treatment approaches is likely feasible and will likely aid in reducing symptom severity and the frequency of problem behaviors.

Our study had several limitations. First, our approach to LPA assigned participants to respective class memberships by choosing each participant's class with the greatest posterior probability generated from the LPA. Posterior probability reflects the probability a participant would belong to a specific class (i.e., classes 1–7), based on observed data. Relative to the LPA approach we used, other approaches have drawbacks, but they can also incorporate methods that actively model measurement error in class assignment, rather than assigning each participant to their most likely class with no error (Elliott et al., 2020). Relatedly, given ASEBA and HSA are self‐report measures, shared method variance could have biased our LPA models; future research would benefit from multi‐informant approaches. Second, our well‐validated, broadband measure of psychopathology only assessed for symptoms of six DSM‐5 disorders, four of which are largely internalizing disorder syndromes. Hence, it is possible our results may not generalize to youth with other comorbid symptoms—including trauma, other externalizing disorders (e.g., substance use), psychotic disorders, and other forms of psychopathology not assessed in our study. Third, given our sample was restricted to participants with psychiatric impairment requiring intensive outpatient therapy or higher, it is possible our findings do not generalize to youth at lower levels of care. Furthermore, our sample included limited racial/ethnic diversity and had low representation among *cis*‐gender males. Relatedly, self‐report variables were standardized using participants' sex assigned at birth, leading to biased estimates of syndrome severity and social‐emotional skills for nonbinary and transgender participants. Finally, we did not systematically collect information about participants’ family income or other indicators of socioeconomic status, making it difficult to further characterize the demographics of our sample. While our sample is one of the biggest and most representative to date on this topic, and reflective of the demographics where our hospitals are located, it is critical for future research to evaluate whether similar associations are found between psychiatric symptoms and socioemotional strengths/challenges among a more broadly representative demographic of youth receiving care in community‐based, outpatient settings. Our division recognizes the large disparity in access to mental health care among historically disinvested populations in our state. We continue to strive to improve access to youth mental healthcare; 85% of our programs accept insurance/co‐insurance, and we will continue to increase the accessibility of our services in the coming years.

### Strengths

Despite these limitations, our study had several notable strengths. First, our large, multi‐site sample (*N* = 926) provides us the ability to detect multiple subgroups, whereas most samples in clinical research are insufficient to achieve this goal. Relatedly, our sample included participants with elevated to extremely elevated clinical severity, an often overlooked population in research due to limited sample sizes and difficulty conducting research in this population. Our multivariate findings extend previous research on this population in community samples (Malti & Noam, 2016; Noam et al., 2012), which found bivariate associations between psychiatric syndromes and socioemotional strengths/challenges.

### Conclusions and future directions

These findings build on previous research (Malti & Noam, 2016; Noam et al., 2012) showing protective social‐emotional patterns are evident not only in school samples, but also among youth in higher levels of clinical care. Moreover, results underscore psychiatric syndrome severity does not map linearly onto social‐emotional functioning. Youth with similar syndromes may report markedly different patterns of strengths and vulnerabilities. Consistent with our findings of heterogeneity in syndrome‐strength profiles, newer neurodevelopmental work suggests not all youth exposed to risk show the same brain outcomes, but timing of stress, pubertal stage, and social environment appear to moderate the impact (Pfeifer & Allen, 2021). As adolescence encompasses both neurobiological maturation and delayed social role transitions (Dahl et al., 2018; Sawyer et al., 2018), examining psychiatric syndromes and social‐emotional strengths together is critical for informing policies and practices that recognize adolescence as a prolonged phase of opportunities as well as vulnerabilities.

Our findings emphasize the clinical utility of measurement‐based care approaches that integrate strengths‐ and resilience‐based frameworks into psychiatric assessment and treatment planning, as differences in social‐emotional development are not observable using syndrome data alone. Hence, we advocate for shifting beyond deficit‐focused models toward more personalized approaches that address risk while also leveraging youths' existing capacities for growth and recovery. For example, each profile identified may be associated with distinct patterns of risk and functioning at school, home, and with peers, and would likely benefit from a tailored treatment approach to address specific syndromes while leveraging inherent strengths. Future studies should seek to identify how impairment and functioning differs between profiles and isolate the mechanisms of change for the socioemotional skills so they can be integrated into existing, evidence‐based treatment for psychiatric disorders.

## AUTHOR CONTRIBUTIONS

**Logan R. Cummings**: Conceptualization; formal analysis; investigation; methodology; project administration; visualization; writing—original draft preparation; writing—review and editing. **Gil Noam**: Methodology; resources; software; visualization; writing—review and editing. **Alessandro S. De Nadai**: Formal analysis; methodology; visualization; writing—original draft preparation; writing—review and editing. **Kathryn Parker**: Data curation; formal analysis; investigation; project administration; visualization; writing—review and editing. **Patricia Allen**: Conceptualization; methodology; visualization; writing—original draft preparation; writing—review and editing. **Julianne G. Wilner.** Investigation; project administration; writing—review and editing. **Jillian M. Russo**: Investigation; project administration; writing—review and editing. **Lauren O. Delman**: Data curation; investigation; project administration; writing—review and editing. **Aishwarya Surendran**: Data curation; investigation; project administration; writing—review and editing. **Emily G. Arnott**: Data curation; investigation; project administration; writing—review and editing. **Dawn E. Sugarman**: Project administration; resources; writing—review and editing. **Alisa Busch**: Project administration; resources; writing—review and editing. **Alexander Kos**: Data curation; project administration; resources; writing—review and editing. **Fairlee C. Fabrett**: Project administration; writing—review and editing. **Ralph Buonopane**: Project administration; resources; writing—review and editing. **Kristen L. Batejan**: Project administration; writing—review and editing. **Erika C. Esposito**: Investigation; project administration; writing—review and editing. **Tian Yu Cheng**: Investigation. **Samuel J. Marquis**: Investigation; writing—review and editing. **Wilson Giles Stecher**: Investigation; writing—review and editing. **Caroline S. Abut**: Investigation; writing—review and editing. **Fiona Ritchie**: Investigation; writing—review and editing. **Jeffrey Zhang**: Investigation; writing—review and editing. **Emily A. Hutchinson**: Investigation; writing—review and editing. **Isabella Kahhale**: Investigation; writing—review and editing. **Hannah Becker**: Investigation; writing—review and editing. **Jennifer T. Sneider**: Writing—review and editing. **Marisa M. Silveri**: Writing—review and editing. **Colin W. Burke**: Writing—review and editing. **Elan French**: Writing—review and editing. **Esther S. Tung**: Writing—review and editing. **Abigail M. Stark**: Writing—review and editing. **Jennifer R. Klix**: Investigation; project administration; writing—review and editing. **Anna B. Mazur**: Investigation; writing—review and editing. **Denice E. Cronin**: Writing—review and editing. **Kayla M. Turner**: Investigation; writing—review and editing. **Melanie R. Harkins**: Investigation; writing—review and editing. **Peter Adams**: Investigation; writing—review and editing. **Willow Clayton**: Investigation; writing—review and editing. **Clare Foster**: Investigation; writing—review and editing. **Dorothea R. Volpp**: Investigation; writing—review and editing. **Emily Gaddy**: Investigation; writing—review and editing. **Florence R. Stout**: Investigation; writing—review and editing. **Alyssa L. Faro**: Investigation; writing—review and editing. **Maria G. Fraire**: Writing—review and editing. **Mary Gambill**: Investigation; writing—review and editing. **Perihan Esra Guvenek‐Cokol**: Writing—review and editing. **Christopher Alpert**: Investigation; writing—review and editing. **Cynthia Kaplan**: Writing—review and editing. **Erin V. Batarseh**: Investigation. **Gillian Galen**: Writing—review and editing. **Kesley A. Hobbs**: Investigation; writing—review and editing. **Lola M. Rodriguez**: Investigation; writing—review and editing. **Lukas A. Turlick**: Investigation; writing—review and editing. **Rhea Agrawal**: Investigation; writing—review and editing. **Sophia E. Nadeau**: Investigation; project administration; writing—review and editing. **Allison P. Falls**: Investigation; writing—review and editing. **Eliot Fearey**: Writing—review and editing. **Daniella H. Levy**: Writing—review and editing. **Ana L. Ibarra**: Investigation; writing—review and editing. **Bryan C. Pridgen**: Writing—review and editing. **Julia G. Luppino**: Investigation; writing—review and editing. **Kelly Molthrop**: Investigation; writing—review and editing. **Louise J. Weidner**: Investigation; writing—review and editing. **Munya Hayek**: Writing—review and editing. **Kayla J. Saucedo**: Investigation; writing—review and editing. **Magdalena S. Batlle**: Investigation; writing—review and editing. **Payton B. Maclean**: Investigation; writing—review and editing. **Rajvi Broker‐Sen**: Writing—review and editing. **Wendy P. Bamatter**: Investigation; writing—review and editing. **Charles Moore**: Writing—review and editing. **Rachel L. Wallace**: Writing—review and editing. **Rae‐Anna Muise**: Writing—review and editing. **Tessa N. Hamilton**: Investigation; writing—review and editing. **Jacqueline B. Sperling**: Writing—review and editing. **Paulina Loo**: Writing—review and editing. **R. Meredith Elkins**: Writing—review and editing. **Michelle C. Silverman**: Writing—review and editing. **Daniel P. Dickstein**: Conceptualization; funding acquisition; methodology; project administration; resources; supervision; visualization; writing—review and editing.

## CONFLICT OF INTEREST STATEMENT

G.N. serves on the board of Partnerships in Education and Resilience, an organization that may be affected by this research. The remaining authors have declared that they have no competing or potential conflicts of interest.

## ETHICAL CONSIDERATIONS

This research was approved by the Mass General Brigham Institutional Review Board (Protocol #2021P002913) on June 16, 2023 All participants provided informed consent/assent.

## Supporting information

Supporting Information S1

## Data Availability

The data that support the findings of this study are available from the corresponding author upon reasonable request.
