# Supplementary material for: Patterns of psychopathology and personal strengths among youth in psychiatric care
Source: JCPP Adv. 2026 Aug 6:e70156. Online ahead of print. doi: 10.1002/jcv2.70156 (PMC13447985; doi:10.1002/jcv2.70156)
Supplement: Supplementary file 1 [file JCV2-9999-e70156-s001.docx]

**Patterns of Psychopathology and Personal Strengths among Youth in Psychiatric Care**

**Supporting Information**

**Table S1.**

*Prevalence of Nonbinary Gender Identities*

|  | *n* | % |
| --- | --- | --- |
| agender | 6 | 0.65 |
| demiboy | 2 | 0.22 |
| demigirl | 5 | 0.54 |
| femboy | 1 | 0.11 |
| femme | 1 | 0.11 |
| genderfluid | 17 | 1.84 |
| genderneutral | 1 | 0.11 |
| genderqueer | 6 | 0.65 |
| girl flux | 1 | 0.11 |
| nonbinary, unspecified | 60 | 6.48 |
| pangender | 3 | 0.32 |
| transfeminine | 2 | 0.22 |
| transmasculine | 6 | 0.65 |
| xenogender | 1 | 0.11 |

**Appendix S1. Latent Profile Analysis and selection of a 7-Class Model**

We began our LPA by inspecting changes in BIC values for 1 to 12 models, presented in Table S2 and plotted in Figure S1. We noticed inflection points (i.e., “scree plot elbow”) in BIC values when comparing model fit between a 4- to 5-class model, as well as when comparing fit between a 7- to 8-class model. Then, we plotted the 4-, 5-, 7-, and 8-class models (Figures S1, S2, S3, and S4, respectively) to determine qualitative interpretability.

**Table S2.**

*Fit Indices for Models with 1-12 Latent Classes*

| # Classes | Log-Likelihood | BIC | Change in BIC | Entropy | Proportion of Sample in Smallest Class |
| --- | --- | --- | --- | --- | --- |
| 1 | -19978.97 | 40162.90 |  | 1 | 100.0% |
| 2 | -18981.33 | 38276.92 | -1885.98 | 0.848 | 36.2% |
| 3 | -18631.26 | 37686.10 | -590.82 | 0.818 | 24.3% |
| 4 | -18287.49 | 37107.87 | -578.24 | 0.826 | 18.4% |
| 5 | -18141.66 | 36925.53 | -182.34 | 0.821 | 13.6% |
| 6 | -18011.93 | 36775.38 | -150.15 | 0.817 | 10.6% |
| 7 | -17859.55 | 36579.93 | -195.45 | 0.832 | 8.2% |
| 8 | -17774.24 | 36518.61 | -61.32 | 0.832 | 8.5% |
| 9 | -17702.60 | 36484.65 | -33.97 | 0.826 | 7.6% |
| 10 | -17631.45 | 36451.65 | -33.00 | 0.825 | 5.6% |
| 11 | -17576.33 | 36450.72 | -0.93 | 0.824 | 5.6% |
| 12 | -17539.59 | 36486.55 | 35.83 | 0.820 | 4.2% |

**Figure S1.**

*4-Class Latent Profile Model*

The 4-class model had two superordinate clusters differentiated by psychopathology severity (2 classes with elevated psychopathology and 2 classes with extremely elevated psychopathology), whose within-cluster classes were characterized by distinct patterns of strengths and resiliencies. We observed a similar pattern for the 5-class solution, except there were 3 classes characterized by extremely elevated psychopathology.

**Figure S2.**

*5-Class Latent Profile Model*

The 7-class solution had 3 clear superordinate clusters differentiated by psychopathology (2 elevated, 2 very elevated, and 3 extremely elevated), whose within-cluster classes were characterized by distinct patterns of strengths and resiliencies. Given the clearly distinguishable superordinate clusters in the 7-class solution, we viewed this as a meaningful improvement in model interpretability over the 4- and 5-class solutions, causing us to remove the 4- and 5-class models from consideration.

**Figure S3.**

*7-Class Latent Profile Model*

We observed a similar pattern for the 8-class solution as the 7-class solution, except an additional class with very elevated psychopathology emerged in the 8-class solution. Upon further inspection, we did not perceive this additional class to be clearly distinguishable from the other classes in the very elevated psychopathology cluster and that the 8-class model provided minimal improvement in interpretability over the 7-class model.

**Figure S4.**

*8-Class Latent Profile Model*

Based on BIC values and qualitative analysis, we preliminarily determined the 7-class model would be the best fit for the data. We then examined model entropy - model entropy was relatively similar across all models (~0.82), indicating similar levels of classification accuracy across models. Finally, we examined the size of smallest class across models. Nylund et al. (2007) recommend the smallest class size in an LPA should be > 10% of the total sample. Compared to the 4- (18.4%) and 5-class (13.6%) models, the 7- (8.2%) and 8-class (8.5%) solutions are smaller than Nylund et al. recommendation. However, given our large sample size (N= 926), we were assured that retaining the 7-class solution reasonably balanced other fit indices (BIC, entropy) and model interpretability with model reliability.

**Table S3.**

*Mean z-score, Standard Errors, and 95% Confidence Intervals for each Indicator Variable, per Profile (plotted in Figure 1)*

| Profile | Variable | ***M*** | *SE* | Upper CI | Lower CI |
| --- | --- | --- | --- | --- | --- |
| *Int-Low Assertiveness/Reflection* | Depression | **1.35** | 0.07 | 1.21 | 1.50 |
|  | Anxiety | 0.89 | 0.06 | 0.77 | 1.01 |
|  | OCD | 0.80 | 0.05 | 0.70 | 0.90 |
|  | Somatic Distress | 0.54 | 0.05 | 0.44 | 0.64 |
|  | ADHD | 0.74 | 0.06 | 0.62 | 0.85 |
|  | Conduct Disorder | 0.53 | 0.06 | 0.42 | 0.64 |
|  | Action Orientation | -0.54 | 0.08 | -0.71 | -0.37 |
|  | Emotion Control | 0.28 | 0.07 | 0.14 | 0.43 |
|  | Assertiveness | -0.90 | 0.09 | -1.08 | -0.72 |
|  | Perseverance | -0.96 | 0.07 | -1.10 | -0.83 |
|  | Optimism | -0.87 | 0.07 | -1.00 | -0.74 |
|  | Reflection | -0.95 | 0.07 | -1.09 | -0.81 |
|  | Trust | 0.00 | 0.08 | -0.15 | 0.15 |
|  | Empathy | 0.03 | 0.08 | -0.13 | 0.19 |
|  | Rel. w/ Peers | -0.10 | 0.08 | -0.26 | 0.07 |
|  | Rel. w/ Adults | -0.06 | 0.07 | -0.19 | 0.08 |
| *Int-Belonging Resilience* | Depression | **1.01** | 0.08 | 0.86 | 1.17 |
|  | Anxiety | 0.84 | 0.08 | 0.68 | 1.01 |
|  | OCD | 0.93 | 0.09 | 0.76 | 1.10 |
|  | Somatic Distress | 0.56 | 0.07 | 0.42 | 0.70 |
|  | ADHD | 0.67 | 0.09 | 0.49 | 0.84 |
|  | Conduct Disorder | 0.41 | 0.08 | 0.25 | 0.57 |
|  | Action Orientation | 0.61 | 0.10 | 0.41 | 0.81 |
|  | Emotion Control | 0.23 | 0.13 | -0.01 | 0.48 |
|  | Assertiveness | 0.52 | 0.10 | 0.31 | 0.72 |
|  | Perseverance | 0.55 | 0.09 | 0.37 | 0.72 |
|  | Optimism | 0.55 | 0.08 | 0.38 | 0.71 |
|  | Reflection | 0.60 | 0.08 | 0.45 | 0.74 |
|  | Trust | **1.12** | 0.11 | 0.90 | 1.33 |
|  | Empathy | 0.95 | 0.08 | 0.80 | 1.10 |
|  | Rel. w/ Peers | 0.77 | 0.09 | 0.58 | 0.95 |
|  | Rel. w/ Adults | **1.06** | 0.07 | 0.92 | 1.20 |
| *CoM- High Empathy* | Depression | **2.31** | 0.05 | 2.21 | 2.41 |
|  | Anxiety | **2.08** | 0.05 | 1.98 | 2.18 |
|  | OCD | **2.02** | 0.06 | 1.90 | 2.13 |
|  | Somatic Distress | **1.30** | 0.07 | 1.17 | 1.43 |
|  | ADHD | **1.09** | 0.06 | 0.96 | 1.22 |
|  | Conduct Disorder | 0.53 | 0.05 | 0.44 | 0.62 |
|  | Action Orientation | 0.08 | 0.08 | -0.07 | 0.23 |
|  | Emotion Control | 0.22 | 0.06 | 0.11 | 0.33 |
|  | Assertiveness | -0.39 | 0.07 | -0.53 | -0.24 |
|  | Perseverance | -0.18 | 0.07 | -0.33 | -0.04 |
|  | Optimism | -0.66 | 0.05 | -0.75 | -0.57 |
|  | Reflection | 0.08 | 0.06 | -0.04 | 0.21 |
|  | Trust | 0.48 | 0.07 | 0.33 | 0.62 |
|  | Empathy | 0.86 | 0.05 | 0.77 | 0.95 |
|  | Rel. w/ Peers | 0.34 | 0.07 | 0.20 | 0.48 |
|  | Rel. w/ Adults | 0.62 | 0.05 | 0.51 | 0.72 |
| *CoM- Low Assertiveness/Reflection* | Depression | **2.46** | 0.06 | 2.35 | 2.57 |
|  | Anxiety | **1.58** | 0.06 | 1.48 | 1.69 |
|  | OCD | **1.70** | 0.05 | 1.60 | 1.80 |
|  | Somatic Distress | **1.07** | 0.07 | 0.95 | 1.20 |
|  | ADHD | **1.42** | 0.07 | 1.29 | 1.54 |
|  | Conduct Disorder | **1.39** | 0.06 | 1.27 | 1.51 |
|  | Action Orientation | **-1.09** | 0.07 | -1.24 | -0.95 |
|  | Emotion Control | -0.25 | 0.06 | -0.37 | -0.12 |
|  | Assertiveness | **-1.05** | 0.08 | -1.21 | -0.88 |
|  | Perseverance | **-1.44** | 0.07 | -1.58 | -1.31 |
|  | Optimism | **-1.66** | 0.05 | -1.76 | -1.56 |
|  | Reflection | **-1.44** | 0.08 | -1.59 | -1.29 |
|  | Trust | -0.91 | 0.06 | -1.04 | -0.79 |
|  | Empathy | -0.91 | 0.08 | -1.07 | -0.75 |
|  | Rel. w/ Peers | **-1.02** | 0.08 | -1.18 | -0.87 |
|  | Rel. w/ Adults | -0.96 | 0.06 | -1.08 | -0.83 |
| *CoH- Assertive* | Depression | **3.27** | 0.08 | 3.12 | 3.42 |
|  | Anxiety | **2.55** | 0.07 | 2.41 | 2.69 |
|  | OCD | **2.79** | 0.07 | 2.65 | 2.93 |
|  | Somatic Distress | **2.32** | 0.10 | 2.12 | 2.52 |
|  | ADHD | **2.20** | 0.07 | 2.06 | 2.34 |
|  | Conduct Disorder | **1.85** | 0.07 | 1.71 | 2.00 |
|  | Action Orientation | 0.14 | 0.09 | -0.03 | 0.32 |
|  | Emotion Control | **-1.17** | 0.08 | -1.32 | -1.02 |
|  | Assertiveness | 0.25 | 0.09 | 0.07 | 0.43 |
|  | Perseverance | -0.65 | 0.10 | -0.84 | -0.46 |
|  | Optimism | **-1.17** | 0.08 | -1.32 | -1.03 |
|  | Reflection | -0.18 | 0.08 | -0.35 | -0.02 |
|  | Trust | -0.40 | 0.09 | -0.58 | -0.21 |
|  | Empathy | 0.71 | 0.07 | 0.57 | 0.85 |
|  | Rel. w/ Peers | -0.12 | 0.10 | -0.31 | 0.07 |
|  | Rel. w/ Adults | 0.14 | 0.08 | -0.02 | 0.30 |
| *CoH- Emotion Control* | Depression | **3.21** | 0.04 | 3.14 | 3.29 |
|  | Anxiety | **2.59** | 0.04 | 2.52 | 2.67 |
|  | OCD | **2.54** | 0.04 | 2.46 | 2.63 |
|  | Somatic Distress | **1.73** | 0.06 | 1.62 | 1.84 |
|  | ADHD | **1.38** | 0.05 | 1.29 | 1.47 |
|  | Conduct Disorder | 0.72 | 0.04 | 0.64 | 0.79 |
|  | Action Orientation | -0.66 | 0.06 | -0.78 | -0.53 |
|  | Emotion Control | 0.19 | 0.04 | 0.11 | 0.28 |
|  | Assertiveness | **-1.24** | 0.06 | -1.35 | -1.13 |
|  | Perseverance | -0.95 | 0.06 | -1.07 | -0.83 |
|  | Optimism | **-1.55** | 0.04 | -1.63 | -1.47 |
|  | Reflection | -0.70 | 0.06 | -0.81 | -0.59 |
|  | Trust | -0.51 | 0.05 | -0.61 | -0.41 |
|  | Empathy | 0.61 | 0.05 | 0.52 | 0.70 |
|  | Rel. w/ Peers | -0.57 | 0.06 | -0.69 | -0.46 |
|  | Rel. w/ Adults | -0.31 | 0.05 | -0.41 | -0.21 |
| *CoH- Low Assertiveness/Reflection* | Depression | **3.79** | 0.07 | 3.65 | 3.93 |
|  | Anxiety | **2.78** | 0.07 | 2.64 | 2.92 |
|  | OCD | **3.03** | 0.07 | 2.90 | 3.17 |
|  | Somatic Distress | **2.47** | 0.11 | 2.26 | 2.68 |
|  | ADHD | **2.44** | 0.09 | 2.26 | 2.62 |
|  | Conduct Disorder | **2.07** | 0.09 | 1.91 | 2.24 |
|  | Action Orientation | -0.95 | 0.11 | -1.17 | -0.73 |
|  | Emotion Control | **-1.17** | 0.09 | -1.35 | -0.99 |
|  | Assertiveness | **-1.26** | 0.11 | -1.48 | -1.04 |
|  | Perseverance | **-1.92** | 0.09 | -2.09 | -1.75 |
|  | Optimism | **-2.22** | 0.06 | -2.34 | -2.10 |
|  | Reflection | **-1.48** | 0.09 | -1.66 | -1.29 |
|  | Trust | **-1.48** | 0.08 | -1.63 | -1.32 |
|  | Empathy | -0.48 | 0.14 | -0.75 | -0.22 |
|  | Rel. w/ Peers | **-1.53** | 0.10 | -1.73 | -1.34 |
|  | Rel. w/ Adults | -0.91 | 0.10 | -1.11 | -0.72 |

*Note.* Mean z-scores > |1| are in bold, indicating symptom severity was clinically elevated or a socio-emotional strength/challenge.

Supplemental Data References

Nylund, K. L., Asparouhov, T., & Muthen, B.O. (2007). Deciding on the number of classes in latent class analysis and growth mixture modeling: A Monte Carlos simulation study. *Structural Equation Modeling: A Multidisciplinary Jounrla, 14(4),* 535-569. <https://doi.org/10.1080/10705510701575396>
